# Anti-Cancer Effects of YAP Inhibitor (CA3) in Combination with Sorafenib against Hepatocellular Carcinoma (HCC) in Patient-Derived Multicellular Tumor Spheroid Models (MCTS)

**DOI:** 10.3390/cancers14112733

**Published:** 2022-05-31

**Authors:** Sojung Han, Ji Yeon Lim, Kyungjoo Cho, Hye Won Lee, Jun Yong Park, Simon Weonsang Ro, Kyung Sik Kim, Haeng Ran Seo, Do Young Kim

**Affiliations:** 1 Department of Internal Medicine, Yonsei University College of Medicine, Seoul 03722, Korea; sjhan@eulji.ac.kr (S.H.); lorry-lee@yuhs.ac (H.W.L.); drpjy@yuhs.ac (J.Y.P.); 2 Uijeongbu Eulji Medical Center, Department of Internal Medicine, Eulji University School of Medicine, Uijeongbu 11759, Korea; 3 Yonsei Liver Center, Severance Hospital, Yonsei University Health System, Seoul 03722, Korea; jylim55@yuhs.ac (J.Y.L.); kyungjoo89@yuhs.ac (K.C.); 4 Department of Genetics and Biotechnology, College of Life Sciences, Kyung Hee University, Yongin 17104, Korea; simonro@khu.ac.kr; 5 Department of Surgery, Yonsei University College of Medicine, Seoul 03722, Korea; kskim88@yuhs.ac; 6 Advanced Biomedical Research Laboratory, Institut Pasteur Korea, Seongnam 13488, Korea

**Keywords:** hepatocellular carcinoma (HCC), multicellular tumor spheroids (MCTS), YAP, TAZ, CA3

## Abstract

**Simple Summary:**

Activation of YAP/TAZ (mediators of Hippo signaling) is associated with the development of liver cancer. The expression level of YAP is known to relate to chemoresistance. However, the therapeutic effect of YAP/TAZ inhibition when combined with sorafenib and conventional chemotherapy in HCC is not known. Recent studies have highlighted the importance of the tumor microenvironment (TME) in chemoresistance. The multicellular tumor spheroid (MCTS) model has emerged as a promising tool for studying cancer drugs as it mimics actual TME. Here, we aimed at assessing the YAP/TAZ expression level of HCCs and identifying the therapeutic effects of CA3 (novel YAP inhibitor) when combined with sorafenib using a patient-derived MCTS model. We established patient-derived MCTS and confirmed that patient-derived MCTS with high YAP/TAZ expression responded more sensitively to the combination therapy (sorafenib with CA3) than MCTS with low or medium YAP/TAZ expression. These findings suggest that the YAP/TAZ inhibitor may serve as a potential therapeutic strategy to enhance sensitivity to sorafenib in HCCs with high YAP/TAZ expression.

**Abstract:**

Purpose: To assess the expression levels of YAP and TAZ in patient-derived HCC tissue and identify the effects of YAP/TAZ inhibition depending on the baseline YAP/TAZ expression when combined with sorafenib using a patient-derived multicellular tumor spheroid (MCTS) model. Methods: Primary HCC cell lines were established from patient-derived tissue. Six patient-derived HCC cell lines were selected according to YAP/TAZ expression on Western blot: high, medium, low. Then, MCTS was generated by mixing patient-derived HCC cells and stroma cells (LX2, WI38, and HUVECs) and YAP/TAZ expression was assessed using Western blot. Cell viability of MCTS upon 48 h of drug treatment (sorafenib, sorafenib with CA3 0.1 µM, and CA3 (novel YAP1 inhibitor)) was analyzed. Results: Out of six patient-derived HCC cell lines, cell lines with high YAP/TAZ expression at the MCTS level responded more sensitively to the combination therapy (Sorafenib + CA3 0.1 μM) despite the potent cytotoxic effect of CA3 exhibited in all of the patient-derived HCCs. Conclusion: Targeting YAP/TAZ inhibition using the novel YAP1 inhibitor CA3 could be a promising therapeutic strategy to enhance sensitivity to sorafenib especially in HCCs with high YAP/TAZ expression in MCTS.

## 1. Introduction

Hepatocellular carcinoma (HCC) is the fifth most common cancer and second leading cause of cancer-related deaths worldwide [1,2]. Primary resection is recommended as the first line of treatment for patients with small solitary HCCs with well-preserved liver function [3,4]. Despite surgical resection of early stage tumors, less than 2 cm and without vascular invasion, the recurrence rate reaches approximately 60% at 5 years [5]. Long term survival is significantly reduced in patients with HCC recurrence after primary resection (5-year survival: 58% vs. 34%) [6]. Interestingly, it is reported that the characteristics of the original tumor determine the outcome after the development of a recurrent tumor within 24 months after resection [6]. Therefore, elucidating the character of the original tumor is imperative for the treatment of a recurrent tumor.

The Hippo signaling pathway Is an important regulator of cell proliferation, organ development, and cell survival [7,8,9]. Yes-associated protein (YAP) and its paralog, transcriptional co-activator with PDZ-binding motif (TAZ), are the major effectors of the Hippo signaling pathway, and they are negatively regulated when Hippo signaling is active [10]. When Hippo signaling is inactive, YAP and TAZ co-activators translocate into the nucleus and interplay with TEA domain family members (TEAD) to form a YAP/TAZ-TEAD complex which activates target genes associated with cell proliferation [11]. In recent years, functional studies have elucidated that YAP is an important oncogene and YAP/TAZ is pervasively activated in human tumors [12]. YAP is overexpressed in 62% of patients with HCC, and YAP levels correlated with decreased survival after resection [13,14]. In addition, YAP is associated with stem cell-like behaviors and epithelial-mesenchymal transition (EMT), both of which are key factors in multi-drug resistance (MDR) to chemotherapy [15,16]. A previous study by Zhou demonstrated that HCC cell lines with chemoresistance exhibited overexpression of YAP, and inhibition of YAP endowed HCC with sensitivity to chemotherapeutic agents in vitro and in vivo [17]. Thus, YAP/TAZ has emerged as a potential therapeutic target in HCC, playing important role in regulating chemotherapeutic sensitivity.

A previous study has proven that combining a YAP inhibitor (Verteporfin) with chemotherapeutic agents (5-fluorouracil, doxorubicin) has effectively overcome chemoresistance in HCC cell lines (BEL/FU) and in vivo [17]. However, in the clinical setting, sorafenib is introduced as the first line systemic therapy in HCC and the therapeutic effect of YAP/TAZ inhibition when combined with sorafenib is not known. In the real world, YAP/TAZ expression levels differ among patients, and the effect of YAP/TAZ inhibition on the different levels of YAP/TAZ expression has not been studied in HCC. CA3 (novel YAP1 inhibitor) is a small molecule which was identified through chemical library screening, and its antitumor activity was studied in esophageal carcinoma [18]. However, CA3 and its antitumor activity in HCC are not known.

The tumor microenvironment (TME) is known to correlate with poor response to chemotherapeutic agents in tumors [19,20]. The TME in HCC consists of cancer and stromal cells, including cancer-associated fibroblasts (CAFs), hepatic stellate cells (HSCs), immune and inflammatory cells, and endothelial cells. When HSCs are activated, they secrete growth factors such as transforming growth factor ß1 (TGF-ß1), connective tissue growth factor (CTGF), and platelet-derived growth factor (PDGF) into the TME, resulting in the activation of cancer-stroma leading to enhanced cancer cell proliferation, excessive ECM synthesis, EMT and invasion, as well as drug resistance [21]. In vitro two-dimensional (2D) co-culture models show tumor-CAF interactions but lack the potential to accurately mimic in vivo TME, and animal models lack the availability of human fibroblasts, limiting their usefulness as preclinical models. Therefore, the multicellular tumor spheroid model (MCTS) has emerged as a promising tool for better mimicking the TME interplaying with HCC. These 3D cultures have the potential to improve cell-based drug screening. Considering the heterogeneity of HCC among patients, investigating drug response using a patient-derived 3D culture may give us valuable information in terms of drug sensitivity.

Here, we investigated the YAP/TAZ expression of patient-derived HCCs and demonstrated the therapeutic effect of the combination of novel YAP inhibitor (CA3) and sorafenib using a patient-derived 3D multicellular tumor spheroid model.

## 2. Materials and Methods

### 2.1. Patients and Tissue Sample

Clinical study protocols were approved by the Institutional Review Board (project number IRB 4-2018-1087) at the College of Medicine, Yonsei University of Korea. Tissue samples of human HCC were obtained after curative surgical resection during January 2020 and September 2020 and processed at the Department of Pathology, Severance Hospital, Yonsei University of Korea. Tissue was immediately fixed in 10% neutral buffered formalin for 4 to 8 h depending on the sample size and was subjected to routine paraffin-embedding protocols.

### 2.2. Cell Lines and Culture Conditions

LX2 cells (human hepatic stellate cells; HSCs) were provided by Dr. Haeng Ran Seo (Institute Pasteur Korea). HUVEC cells (human umbilical vein endothelial cells) were obtained from Lonza (Basel, Switzerland). WI38 cells (human fibroblast cell line) were obtained from the Korean Cell Line Bank. The cells were maintained at 37 °C in a humidified atmosphere (5% CO_2_/95% air). LX2 cells were cultured in Dulbecco’s Modified Eagle Medium (DMEM; Gibco, Grand Island, NY, USA) supplemented with heat-inactivated 10% fetal bovine serum (FBS; Gibco, Grand Island, NY, USA) and 1× penicillin (Welgene, Gyeongsan, Korea) (Complete media). HUVEC cells were maintained in Medium 200 (Gibco, USA) supplemented with 1× penicillin (Welgene, Gyeongsan, Korea), 1× LSGS (Gibco, USA) and heat-inactivated 10% FBS. For WI38 cells, Roswell Park Memorial Institute medium (RPMI 1640; Gibco) supplemented with 1× penicillin (Welgene, Gyeongsan, Korea), and 10% heat-inactivated FBS was used. Primary HCC cells were maintained in DMEM/F12 (Gibco, USA) supplemented with 1× penicillin (Welgene, Gyeongsan, Korea), 1× GlutaMmax (Gibco, USA), 1× Primocin (Invitrogen, Carlsbad, CA, USA), and 10% heat-inactivated FBS.

### 2.3. Primary Culture of HCCs

Immediately after surgery, a portion of the tumor was immersed in Hanks balanced salt solution (HBSS; Gibco) and was transported from the operating room at 0 °C to the laboratory. The specimens were collected under sterile conditions and rinsed 2–3 times with HBSS free of calcium and magnesium to remove blood. After removal of blood, single-cell suspensions were prepared from liver tissue specimens using a GentleMACS™ Dissociator (Miltenyi Biotec, Bergisch Gladbach, Germany). The resultant was filtered through a 100 μm-nylon filter (BD Falcon, Franklin Lakes, NJ, USA) and centrifuged at 50× *g* for 2 min at 4 °C to obtain hepatocytes. The pellet was washed twice in HBSS containing 0.005% DNase. The final cell suspensions were cultured onto collagen-coated T25 flasks (BD Falcon) in F12/DMEM (Gibco), supplemented with 20% FBS, 1% NEAA, 1% glutamine, and 1% P/S at 37 °C in a humidified 5% CO_2_ incubator. The medium was changed 24 h after seeding to remove dead cells and debris. When confluence reached 70–80%, the cells were re-plated using a 1:1 mixture of DMEM medium and F12/DMEM with supplements. After five passages, the cells were grown on DMEM medium supplemented with 10% FBS and 1% P/S. Confluent cells were trypsinized, counted and split 1:3–1:5 at every passage. Once cell lines were maintained over 30 passages, they were collected and stored in liquid nitrogen.

### 2.4. Generation of Multicellular Tumor Spheroids (MCTS)

To generate MCTS, cells suspended in complete medium were seeded at a density of 6 × 103 cells/well in 96-well ultra-low-attachment (ULA) round-bottom microplates (Sumitomo Bakelite Co., Tokyo, Japan, PrimeSurface, MS-9096UZ). Mixed-cell spheroids were generated by seeding LX2 cells, HUVEC cells, WI38 cells, and patient-derived primary HCCs at a 1:1:1:3 ratio (1000: 1000: 1000: 3000) in ULA plates. The cells were cultured for 3 days at 37 °C in a humidified atmosphere (5% CO_2_/95% air) without daily media changes for drug treatment.

Once MCTS were generated, images of the generated MCTS were captured in a 10 μm stack using an inverted microscope (IX71; Olympus, Tokyo, Japan) with a 10× objective. Then, MCTS were seeded with drugs for 48 h. Images of MCTS with drug treatment were captured using an inverted microscope (IX71; Olympus, Tokyo, Japan) with a 10× objective.

For the preparation of histological sections, generated MCTS were fixed in 4% paraformaldehyde (Biosesang, Korea) and cut into 5 μm thick sections using a microtome and were mounted on glass slides. The slides were stained with hematoxylin and eosin (H&E). Immunohistochemical staining of paraffin slides of MCTS was performed in order to validate the origin of consisting cells of MCTS: α-SMA (marker for hepatic stellate cells, LX2), fibronectin (marker for fibroblasts, WI 38), CD34 (marker for endothelial cells, HUVEC). Representative images were taken under a microscopy with a 200× objective.

### 2.5. Drug Response Assay

Sorafenib (Nexavar^®^) and CA3 were purchased from Selleckchem (Houston, TX, USA). Stock solutions of sorafenib and CA3 were prepared in 100% DMSO and were stored at −20 °C. All drugs were diluted to different concentrations in culture media. For drug exposure, MCTS were incubated in drug-containing media for 48 h without media change. For drug combination, agents were given simultaneously at predetermined concentrations. After drug treatment, MCTS in each well was pipetted smoothly until being dissociated to single cells and was transferred to a standard white assay plate for cell viability assay. Equivalent volume of CellTiter-Glo^®^ 3D Reagent (Promega, Germany) was added. The plates were shaken for 5 min and luminescence was recorded 30 min after reagent addition. Microplate luminometer (Berthold Technologies, Bad Wildbad, Germany, Centro XS3 LB960) was used.

### 2.6. Protein Extraction and Western Blot Analysis

In order to detect various proteins from cells and spheroids, a proper amount of samples were homogenized and digested in 1× RIPA buffer (Cell Signaling, Denver, MA, USA) which includes 1 mM PMSF (Fluka, Switzerland), 2 g/mL Aprotinin (Sigama, Steinheim, Germany), 1 mM DTT(Invitrogen, Carlsbad, CA, USA) and phosphatase inhibitor cocktail solution (GenDEPOT, Barker, TX, USA) on ice for 1 hr. After the digestion, samples were centrifuged for 25 min at 14,000 rpm in cold microcentrifuge. Then supernatants were collected for use. Western blot experiments were performed following the standard protocol. The following primary antibodies were purchased: anti-YAP/TAZ (8418, Cell Signaling) and anti-EpCAM (ab71916, Abcam). Anti-EpCAM was used and for internal control, an anti-GAPDH antibody (2118, Cell Signaling) was used. Anti-rabbit IgG-HRP (A0545, Sigma) was used as the secondary antibody. Specific bands were detected using the enhanced chemiluminescence (ECL) Western blot detection system (Amersham Pharmacia Biotech, Piscataway, NJ, USA).

### 2.7. Immunohistochemistry

Paraffin sections were deparaffinized in xylene and rehydrated through a gradual decrease in ethanol concentration. The antigen epitopes were then unmasked using sodium citrate buffer (pH 6.0). Subsequently, the sections were incubated overnight at 4 °C using primary antibodies. The following primary antibodies were used: anti-YAP/TAZ (1:200, 8418, Cell Signaling), anti-α-SMA (1:200, ab5694, Abcam), anti-fibronectin (1:100, ab2413, Abcam), CD34(1:100, ab81289, Abcam). After primary incubation, sections were incubated with the appropriate biotinylated secondary antibodies (Vector Laboratories, Burlingame, CA, USA) followed by treatment with freshly prepared DAB substrates (Vector Laboratories). Sections were lightly counter-stained with hematoxylin and mounted.

### 2.8. Immunocytochemistry

To validate the primary cells, cells were fixed with 4% paraformaldehyde (Biosesang, Korea) for 10 min at room temperature, permeabilized with 0.1% Triton X-100 (Sigma-Aldrich, St. Louis, MO, USA) in Dulbecco’s phosphate-buffered saline (DPBS; Welgene) for 30 min at room temperature, and then washed three times with DPBS. The following primary antibodies were used: goat monoclonal anti-human serum albumin (A80-229A, 1:250, Bethyl laboratories, TX, USA), mouse monoclonal anti-human Hep-Par 1(OCH1E5, 1:250, Cell Marque, Darmstadt, Germany), and mouse monoclonal anti-alpha fetoprotein (ab3980, 1:250, abcam, Waltham, MA, USA). Samples were incubated with the primary antibodies for 16 h at 4 °C and then washed for 10 min three times with DPBS. The secondary antibodies used for staining were donkey anti-mouse IgG conjugated with Alexa^®^ Fluor 488 (A-21202, Invitrogen, Eugene, OR, USA) and donkey anti-goat IgG conjugated with Alexa^®^ Fluor 647 (A32849, Invitrogen). Samples were then incubated with secondary antibodies for 1 h at room temperature in the dark and washed for 10 min five times with DPBS. For nuclei staining, cells were incubated with DAPI (D9542-10MG, Sigma-Aldrich, St.Louis, MO, USA) for 10 min at room temperature in the dark and washed with PBS twice quickly. All fluorescence images were obtained using the LSM 700 (Zeiss, Oberkochen, Germany).

### 2.9. Statistical Analysis

Statistical analyses were mainly performed using GraphPad Prism Software (GraphPad, La Jolla, CA, USA), using unpaired two-tailed Student’s t-test and Fisher exact test (for immunohistochemistry). *p* values less than 0.05 were considered statistically significant, and all tests were two-sided.

## 3. Results

### 3.1. Clinical, Pathological Features of HCC

In order to develop patient-derived MCTS for anti-cancer drug treatment, primary HCC tumors were isolated from liver resection specimens of liver cancer patients. The baseline characteristics of the ten patients with HCC are summarized in Table 1. The median age was 63.2 years, and there was a male predominance (8/10, 80%). Most of the cases were related to HBV infection (6/10, 60%) and rarely to HCV infection (1/10, 10%). Half of the cases (5/10, 50%) had cirrhosis. A moderate grade of differentiation of a major component (major differentiation) was most common (8/10, 80%), and both tumor marker levels (AFP, PIVKA-II) were relatively low except one case (Patient No. #8) which showed poor differentiation in both major component and the highest histologic grade (worst pathology). The median tumor size was 3.5 cm (1.6–6.2), half of the cases (50%, 5/10) showed vascular invasion, and majority of the cases had single tumor (9/10, 90%). 

### 3.2. Different YAP/TAZ Expression among HCC Tissue

**Figure 1:**
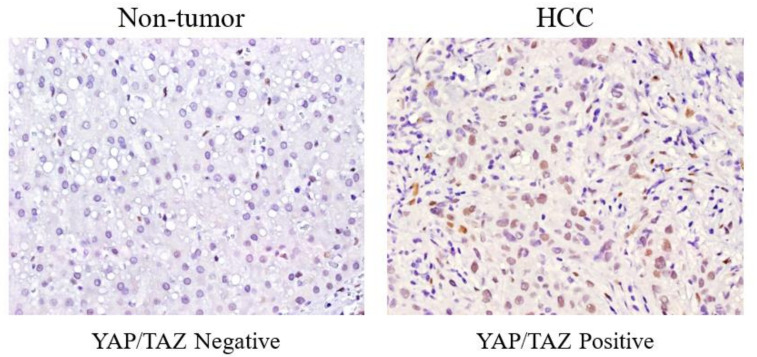
Comparison of YAP/TAZ expression between HCC and non-tumor using immunohistochemistry. Immunohistochemical expression of YAP/TAZ in tissues from HCC patients and adjacent nontumor tissues. Yellowish staining of nucleus demonstrates YAP/TAZ immunostaining, and this was shown as positive in tumor tissue. Adjacent nontumor tissue did not show YAP/TAZ immunostaining. Representative images were taken under a microscope (×400).

Based on a previous study of YAP/TAZ in HCC, we analyzed YAP/TAZ expression between HCC tissue and paired para-tumor liver tissue using immunohistochemistry (IHC). YAP/TAZ expression was observed in primary HCC tissue but not in paired para-tumor liver tissue (Figure 1).

**Figure 2:**
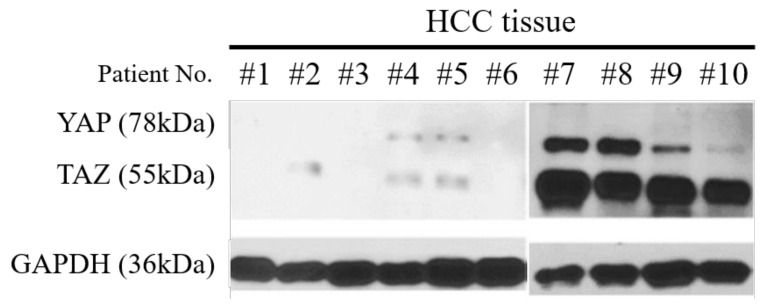
Western blot showing YAP/TAZ expression among HCC tissue. Lysates of HCC tissue were analyzed by Western blotting with anti-YAP/TAZ and anti-GAPDH (control) antibodies. For the original Western blots, see Supplementary Figure S1.

The expression of YAP/TAZ among 10 different patient-derived primary HCC tissue was observed using Western blot (Figure 2). Patient No. #7, #8 showed the highest YAP/TAZ expression, whereas patient no. #1, #3, #6 showed the lowest YAP/TAZ expression. From these, we selected six patient-derived HCC cell lines which are representative of YAP/TAZ expression level as high, medium, and low. These six cell lines were classified into three subgroups according to YAP/TAZ expression level in HCC tissue in order to investigate drug response according to the degree of YAP/TAZ expression; High YAP/TAZ group (Patient No. #7, #8), Medium YAP/TAZ group (Patient No. #4, #5), and Low YAP/TAZ group (Patient No. #3, #6).

**Figure 3:**
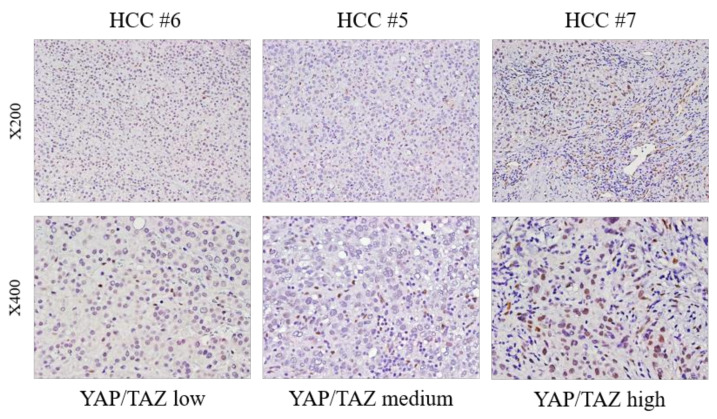
Comparison of YAP/TAZ expression among different primary HCC tissue. Immunohistochemical expression of YAP/TAZ in tissues from HCC patients. Yellowish staining of nucleus demonstrates positive immunostaining of YAP/TAZ. YAP/TAZ positivity was assessed according to the number of positively stained cells: score 0 (<5%), score 1 (6–25%), score 2 (26–50%), score 3 (51–75%), score 4 (>75%). HCC #7 presented with YAP/TAZ score 3 (high), whereas HCC #5 and HCC #6 showed YAP/TAZ score 1 and score 0 respectively. Representative images were taken under a microscope (×200: first row; ×400: second row).

Immunohistochemical staining of YAP/TAZ of HCC tissue was conducted in order to compare YAP/TAZ expression among different HCC tissue. High YAP/TAZ expression was observed in high YAP/TAZ group, whereas medium and low YAP/TAZ expression was observed in medium and low YAP/TAZ group accordingly (Figure 3). YAP/TAZ expression level assessed by Western blot correlates with YAP/TAZ IHC staining in HCC tissue.

### 3.3. Characterization of Primary Cultured HCC Cells as Original HCC

**Figure 4:**
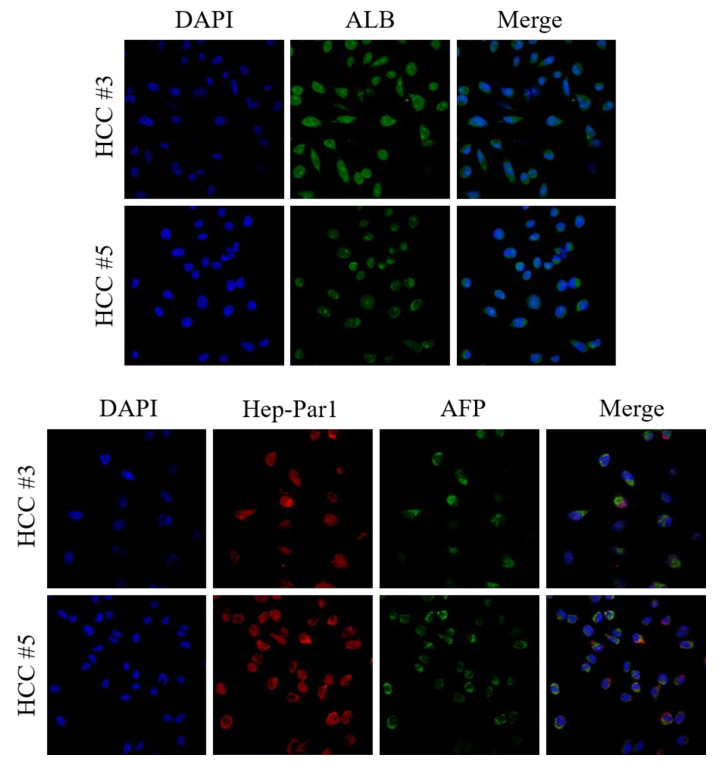
AFP, albumin, and Hep-Par1 immunocytochemical staining of primary HCC cells. AFP, albumin, and Hep Par-1 immunostaining of HCC cell lines were done to examine the cellular origin of primary HCC cells. Representative images were taken under confocal microscopy with a 200× objective.

To define whether primary HCC cell lines maintained the original characteristics of HCC, we performed immunostaining with the hepatocyte-specific markers Hepatocyte Specific Antigen (Hep Par-1), alpha fetoprotein (AFP), and albumin (ALB) (Figure 4). Hep par-1, AFP, and albumin were all expressed in all of the HCC cell lines and expression did not show much difference among all of the patient-derived cell lines.

### 3.4. Generation of MCTS Model Using Patient Derived HCC Cell Lines

The origin of primary HCC cell lines obtained from patients was proven as HCC as mentioned previously. In order to study anti-cancer treatment using a culture method which mimics characteristics of HCC in vivo, we generated multicellular tumor spheroid (3D). For stromal cells in MCTS, LX2 (human hepatic stellate cells, HSCs), WI38 (human fibroblasts), and HUVECs (Human Umbilical Vein Endothelial Cells) were used.

**Figure 5:**
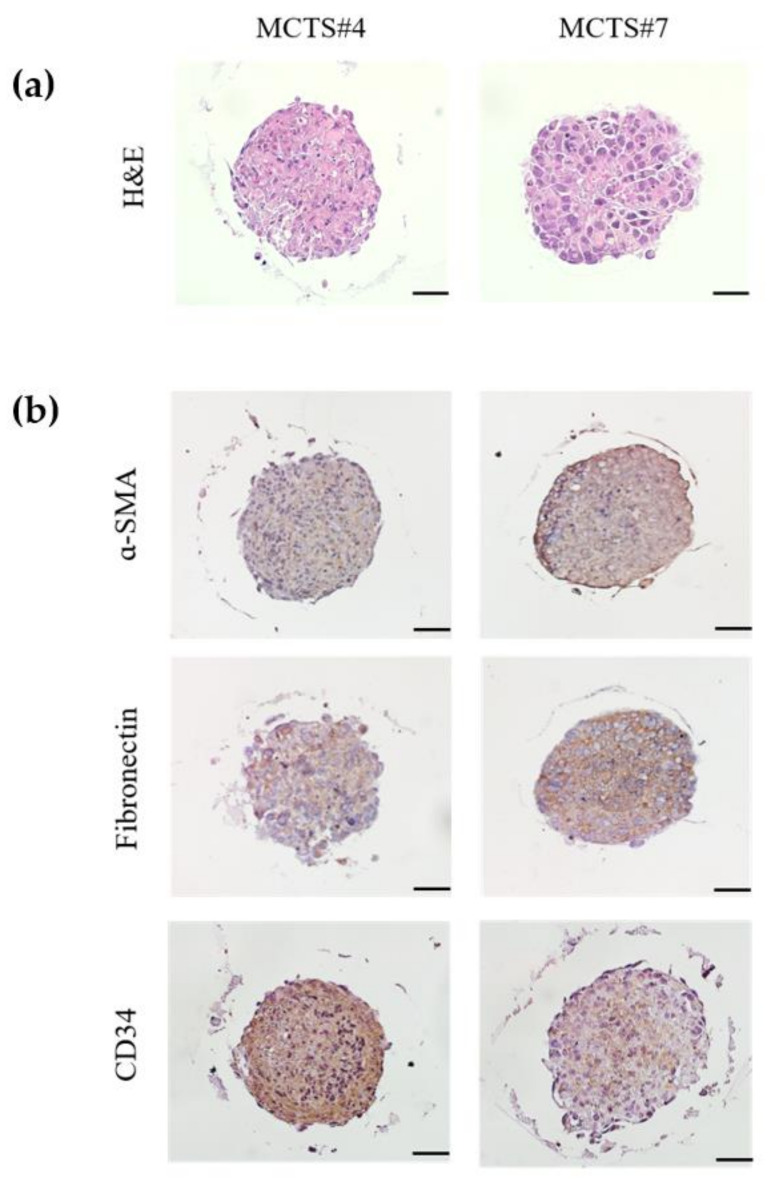
Establishment of MCTS model using patient-derived HCCs and stromal cells. (**a**) Hematoxylin and eosin staining of MCTS; (**b**) Immunohistochemical analysis of α-SMA (marker for hepatic stellate cells, LX2), fibronectin (marker for fibroblasts, WI38), and CD34 (marker for endothelial cells, HUVEC) of consecutive sections of the MCTS model generated from patient-derived primary HCC cells cocultured with human stromal cells (LX2, WI38, and HUVEC). Representative images taken under a microscope (×200). Scale bar, 50 µm.

MCTS paraffin-sections were stained with Hematoxylin and Eosin stain (Figure 5A). Immunohistochemical analysis of MCTS paraffin-sections was performed in order to identify the origin of stromal cells used in MCTS (Figure 5B).

### 3.5. Comparison of YAP/TAZ Expression between Two Different Cultured System: Monolayer (2D) and MCTS (3D) Using Patient-Derived Primary HCC Cells

**Figure 6:**
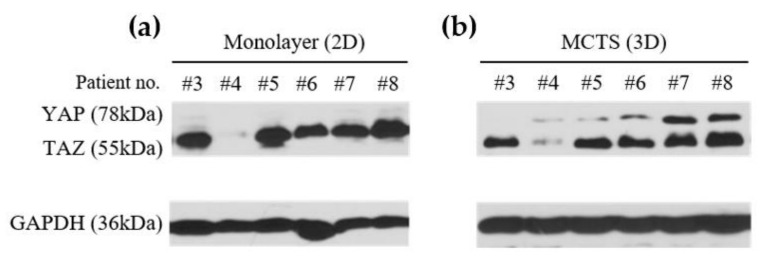
Comparison of YAP/TAZ expression using western blot between monolayer HCCs (2D) and MCTS (3D). For the original Western blots, see Supplementary Figure S1. (**a**) Different expression of YAP/TAZ were assessed by Western blot analysis using monolayer patient-derived HCCs (2D). (**b**) Lysates of MCTS derived from patient-derived HCCs were analyzed by Western blotting with anti-YAP/TAZ and anti-GAPDH (control) antibodies.

In order to compare drug sensitivity according to different YAP/TAZ levels in HCCs, we selected six primary HCC cell lines with different YAP/TAZ levels as mentioned previously. We have generated MCTS using patient-derived HCC cell lines by co-culturing primary HCC cell lines and three types of stromal cells: LX2 (human hepatic stellate cells, HSCs), WI38 (human fibroblasts), and HUVECs (Human Umbilical Vein Endothelial Cells). Western blot was performed to compare the differences of YAP/TAZ expression depending on the culture method; monolayer (2D) and MCTS (3D) (Figure 6). Monolayer HCCs showed TAZ expression on Western blot, whereas MCTS (3D) of patient-derived HCCs showed YAP expression, which was also shown in HCC tissue.

**Figure 7:**
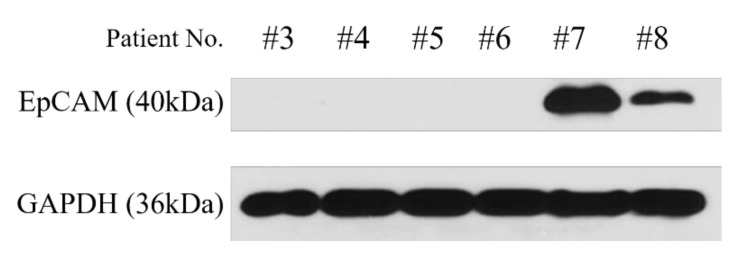
EpCAM expression using Western blot in MCTS. For the original Western blots, see Supplementary Figure S1. Different expression of EpCAM was assessed by Western blot analysis in MCTS.

EpCAM (epithelial cell adhesion molecule) expression was assessed using Western blot in MCTS to look for stemness associated with high YAP/TAZ expression (Figure 7). High YAP/TAZ expressed MCTS No. #7, #8 showed strong EpCAM expression whereas no expression was observed from MCTS with medium or low YAP/TAZ expression.

### 3.6. Comparison of Sensitivity to Anti-Cancer Drugs (Sorafenib, CA3, Combined Therapy) in MCTS

**Figure 8:**
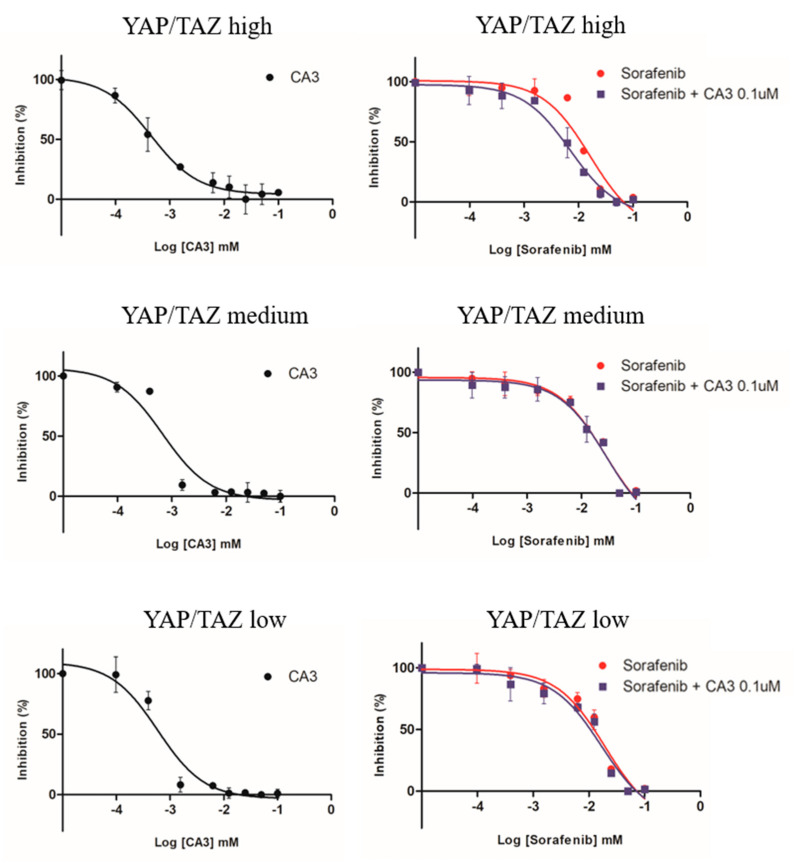
Comparative dose response curves using MCTS. MCTS were treated with CA3 at the indicated concentrations for 48 h. The dose response curve of CA3 monotherapy was presented as black full circles. MCTS were treated with sorafenib alone or sorafenib combined with CA3 0.1 μM at the indicated concentrations for 48 h. Dose response curves of sorafenib (red full circles) and sorafenib combined with CA3 0.1 μM (blue full squares) are presented.

In this study, we sought to investigate the effect of YAP/TAZ inhibitor (CA3) when combined with sorafenib among different YAP/TAZ expressing patient-derived HCC using MCTS. We compared the half maximal inhibitory concentrations (IC50) of sorafenib monotherapy, CA3 monotherapy, and combination therapy (sorafenib combined with CA3 0.1 μM) in MCTS. We have classified MCTS into three subgroups according to different YAP/TAZ expression level according to Western blot: YAP/TAZ high, YAP/TAZ medium, and YAP/TAZ low. Table 2 summarizes the IC50 of anti-cancer drugs in MCTS and Figure 8 shows corresponding dose response curve. All MCTS showed sensitivity to CA3 monotherapy. With regard to combination therapy, YAP/TAZ high MCTS was more sensitive to a combination of sorafenib and CA3 0.1 μM than rest of the other MCTS (YAP/TAZ medium, YAP/TAZ low). YAP/TAZ medium MCTS and YAP/TAZ low MCTS did not show much difference between combination therapy (sorafenib and CA3 0.1 μM) and sorafenib monotherapy.

**Figure 9:**
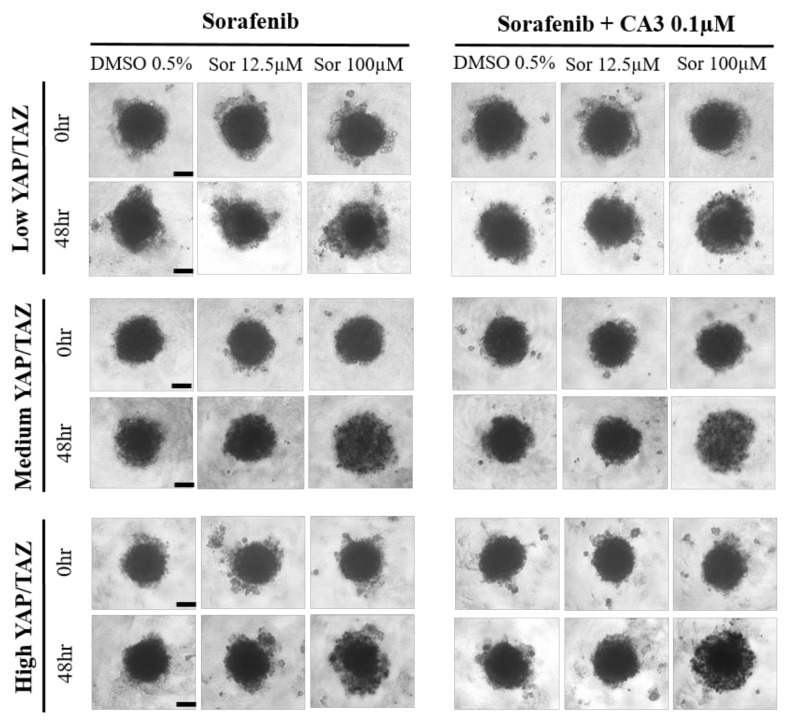
Morphology of generated patient-derived MCTS and serial morphologic changes upon drug treatment. Images of spheroids of MCTS were obtained using an inverted microscope (IX71; Olympus, Tokyo, Japan) with a 10× objective. Scale bar, 20 μm.

Figure 9 shows the morphology of the generated patient-derived MCTS after 48 h of co-culture and the interval change of the morphology of MCTS following drug treatment. Lysis of cells surrounding the surface border of MCTS was shown upon drug treatment. Morphologic changes between sorafenib monotherapy and combination treatment (Sorafenib + CA3 0.1 µM) did not show much difference among different YAP/TAZ level expressed MCTS.

## 4. Discussion

HCC takes up 90% of hepatic malignancy and is the second leading cause of cancer-related deaths worldwide. Treatment at an early stage includes surgical resection or ablative therapy but recurrence rate is high—up to 70% within 5 years. Early recurring tumors have a poor prognosis and are associated with the original primary tumor. First-line systemic therapeutic treatment is a multi-tyrosine kinase inhibitor, sorafenib. Although several systemic therapeutic agents other than sorafenib have been introduced, therapeutic efficacy is still suboptimal. Therefore, novel therapeutic strategies are needed to improve survival in patients with HCC.

YAP/TAZ has emerged as a therapeutic target in many types of cancer including HCC, as the aberrant activation of YAP/TAZ has been implicated in cancer progression and development. Mounting evidence suggests that the deregulation of Hippo signaling and the activation of its co-activators YAP/TAZ lead to multi-drug resistance in many types of cancer, including HCC [22,23,24]. There have been attempts to overcome multi-drug resistance by combining with YAP/TAZ inhibitors in HCC. Song et al. showed that BEL/FU HCC cell lines resistant to 5-Fluorouracil regain sensitivity when combined with Verteporfin (YAP inhibitor). A novel YAP inhibitor, CA3, has shown potent inhibitory effects on YAP/TEAD transcriptional activity and synergistic inhibition when combined with 5-FU especially in high YAP and resistant cells in Esophageal Adenocarcinoma [18]. However, YAP/TAZ inhibition combined with sorafenib in HCCs with different YAP/TAZ expression has yet to be studied. Given that sorafenib is the first-line systemic therapy in HCC at a later stage, it is imperative to investigate whether the sensitivity of sorafenib could be enhanced by YAP/TAZ inhibition depending on YAP/TAZ expression levels.

Besides YAP/TAZ signaling, the tumor microenvironment (TME) plays an important role in cancer progression, cellular differentiation, and therapeutic efficacy [19,25,26]. Recent studies revealed that tumorigenesis relies on the interactions between tumor cells and the surrounding stroma in HCC [26,27,28]. The MCTS model (3D) has emerged as a useful tool for anti-cancer research to implement tumor heterogeneity and complex TME in vitro. In this study, we generated MCTS using patient-derived HCC cells to mimic the characteristics of the HCCs in vivo. To better understand the effect of YAP/TAZ inhibition when combined with sorafenib depending on different YAP/TAZ expressed HCCs, we conducted a drug response test of sorafenib and sorafenib combined with CA3 (YAP 1 inhibitor) using patient-derived MCTS with different YAP/TAZ levels.

The results of this study showing high YAP/TAZ expression in HCC tissue assessed by IHC staining correlate with those of a previous study presenting YAP expression in HCC but not in normal liver tissue [29]. IHC staining of YAP/TAZ was localized in the nucleus of HCC tissue suggesting activated YAP/TAZ and suppressed Hippo signaling. Western blot analysis of YAP/TAZ expression in HCC tissue showed a higher expression of TAZ than YAP expression and expression level does not correlate with the degree of differentiation which is similar to the previous study [15].

YAP/TAZ expression levels from patient-derived HCC cell lines (2D culture) also showed a higher expression of TAZ than YAP expression and this is confirmed by Hayashi et al. [15]. Monolayer HCC cells did not express YAP expression which was shown in both HCC tissue and the MCTS model. While only HCC cells are cultured in the monolayer 2D culture system, HCC cells and stromal cells are co-cultured in the MCTS model. Therefore, the discrepancy in YAP expression according to culture methods may be attributable to the interaction between cancer cells and stromal cells. This stresses the importance of the MCTS model which mimics the actual tumor microenvironment leading to a different protein expression pattern in YAP/TAZ. Song et al. reported the role of hepatic stellate cells (HSCs) in MCTS as chemoresistance, and migration, which exerts its effect by interaction between HSCs and HCC cells [30]. Cancer-associated fibroblasts (CAFs) are the major component of TME and YAP function is critical for the establishment and maintenance of CAFs. A growing body of evidence suggests that YAP-driven ECM stiffening creates a positive feedback loop and sustains YAP activation [31,32,33]. This explains the increased expression of YAP levels in MCTS compared to original HCCs in monolayer (2D) with low YAP expression.

YAP is known to promote multi-drug resistance in HCC, and a blockade of YAP using verteporfin conferred the sensitivity of chemoresistant HCC cell lines to the chemotherapeutic agent [17]. This study chose a novel YAP inhibitor, CA3, as this is a potent YAP inhibitor demonstrated in cancer stem cell (CSC)-enriched esophageal cancer [18]. Our results showed that MCTS with high YAP/TAZ expression endowed sensitivity to sorafenib when CA3 was added. Interestingly, MCTS with low to medium YAP/TAZ expression did not show much difference to the combination effect of CA3 despite its sensitivity to CA3 monotherapy.

Targeting YAP and TAZ is presumed to be safe, as deletion of YAP and TAZ did not present a deleterious effect on liver homeostasis. Therefore, YAP inhibition using CA3 could be a feasible solution for treating HCC in real practice. The expression level of YAP/TAZ in HCC in patients could be assessed using tissue microarray. Patients presenting with high YAP/TAZ expression could be selected for combined CA3 treatment with conventional sorafenib. The generation of patient-derived MCTS and drug treatment may provide informative results for drug application using CA3. This could possibly lead to anti-cancer therapy tailored to individual patients. Further studies are warranted for the correlation of YAP/TAZ expression level among different modalities: estern blot analysis, tissue microarray. In addition, drug application using CA3 could be verified using HCC organoids in the future given that tumor organoids represent more organized and structural units than MCTS.

This study demonstrated that CA3 was effective in reducing chemoresistance to sorafenib in MCTS, which did not show a shift to predominant YAP expression upon TAZ depletion. This suggests that the YAP inhibitor was effective when YAP and TAZ expression were well balanced. Therefore, CA3 might be a possible potential therapeutic agent to reduce the chemoresistance of sorafenib in patients presenting with balanced YAP and TAZ expression in MCTS (3D).

## 5. Conclusions

In this study, different degrees of YAP/TAZ expressed MCTS models have been established using patient-derived HCC cell lines. MCTS with high YAP/TAZ expression showed an increased response to sorafenib when combined with CA3, whereas MCTS with low or medium YAP/TAZ expression did not show any differences in drug sensitivity to sorafenib when combined with CA3.

## Figures and Tables

**Table 1:** Baseline characteristics of patients related to HCC.

Patient No.	Age/Sex	Etiology	Cirrhosis	Major Differentiation	Worst Pathology	Tumor Size (cm)	Number of Tumor	Vascular Invasion	AFP(ng/mL)	PIVKA-II (mAU/mL)
#1	65/M	non-viral	None	Well	Moderate	3.3	1	Present	2.5	42
#2	50/M	HBV	None	Moderate	Poor	3.2	1	not present	8.5	19
#3	71/M	non-viral	Cirrhosis	Moderate	Moderate	1.6	1	not present	4	31
#4	62/F	HBV	None	Moderate	Moderate	6.2	1	not present	2.2	73
#5	78/M	HCV	Cirrhosis	Moderate	Poor	3.7	1	Present	18.2	21
#6	53/M	HBV	Cirrhosis	Moderate	Poor	3.8	2	not present	7.4	49
#7	57/M	HBV	None	Moderate	Moderate	3.1	1	Present	1.9	77
#8	59/M	HBV	Cirrhosis	Poor	Poor	6	1	Present	31.3	445
#9	69/F	HBV	Cirrhosis	Moderate	Poor	4.4	1	Present	1.6	18
#10	68/M	non-viral	None	Moderate	Moderate	1.8	1	not present	5	71

AFP: alpha feto protein; PIVKA-II: Protein induced by vitamin K absence or antagonistry. Major differentiation: Histologic grade of major component; Worst pathology: the highest histologic grade).

**Table 2:** Half maximal inhibitory concentrations (IC50) of anti-cancer drugs in patient-derived MCTS.

MCTS	CA3	Sorafenib + CA3 0.1 μM	Sorafenib
YAP/TAZ high	0.45 μM	9.4 μM	16.4 μM
YAP/TAZ medium	0.46 μM	27.4 μM	28.7 μM
YAP/TAZ low	0.38 μM	16.7 μM	19.4 μM

## Data Availability

All data generated during this research are presented in the manuscript.

## Supplementary Materials

The following supporting information can be downloaded at: https://www.mdpi.com/article/10.3390/cancers14112733/s1, Figure S1: Expanded, uncropped original Western blot panels from Figure 2, Figure 3, Figure 4, Figure 5, Figure 6 and Figure 7.
