# Supplementary material for: Anti-Cancer Effects of YAP Inhibitor (CA3) in Combination with Sorafenib against Hepatocellular Carcinoma (HCC) in Patient-Derived Multicellular Tumor Spheroid Models (MCTS)
Source: Cancers (Basel). 2022 May 31;14(11):2733. doi: 10.3390/cancers14112733 (PMC9179573; doi:10.3390/cancers14112733)
Supplement: Supplementary file 1 [file cancers-14-02733-s001.zip › cancers-1701696-supplementary.pdf]

Figure 2.

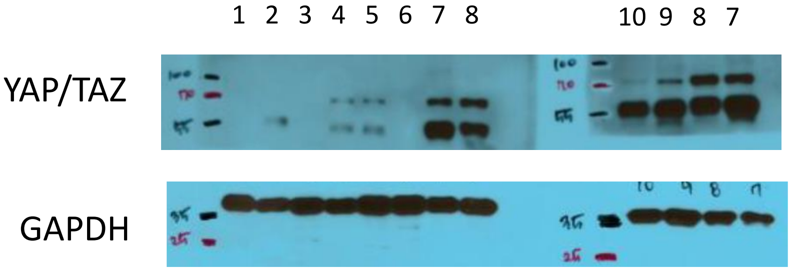

Figure 6A.

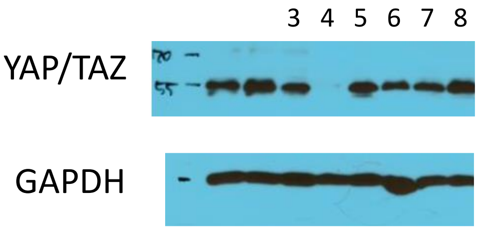

Figure 6B.

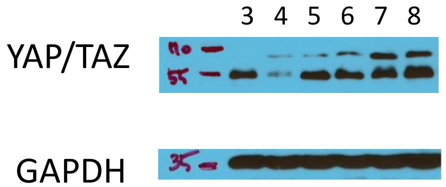

Figure 7.

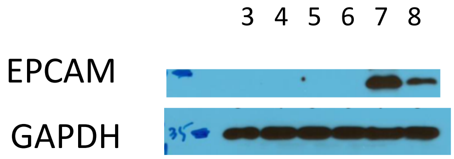

Figure S1. Expanded, uncropped original western blot panels from Figures 2-7.
